# Non-contrast coronary artery wall and plaque imaging using inversion-recovery prepared steady-state free precession

**DOI:** 10.1186/s12880-015-0071-2

**Published:** 2015-07-25

**Authors:** Takeshi Ishimoto, Yasuyo Taniguchi, Tosiaki Miyati, Momoe Kawakami, Masaru Ishihara

**Affiliations:** Division of Health Sciences, Graduate School of Medical Science, Kanazawa University, 5-11-80, Kodatsuno, Kanazawa, 920-0942 Japan; Department of Cardiology, Hyogo Brain and Heart Center, 520, Saisho-ko, Himeji, Hyogo 670-0981 Japan; Department of Radiology, Hyogo Brain and Heart Center, 520, Saisho-ko, Himeji, Hyogo 670-0981 Japan

**Keywords:** Coronary, Plaque, MRI, SSFP, Inversion recovery

## Abstract

**Background:**

The objective of this study was to investigate whether three-dimensional (3D) single inversion-recovery prepared steady-state free precession (IR-SSFP) could characterize the coronary artery wall.

**Methods:**

IR-SSFP was scanned on a 1.5-T MR scanner with a five element cardiac coil. One hundred and twenty-one subjects with known or suspected coronary artery disease who had undergone X-ray coronary angiography (XCA) underwent coronary artery wall imaging using IR-SSFP sequences. In each coronary segment, the detection of the coronary wall was categorized, and contrast (signal of plaque minus signal of blood in the aorta divided by the signal of plaque plus signal of blood in the aorta) was calculated.

**Results:**

422 of 517 segments (82 %) were successfully visualized, and the detection scores tended to be higher at the proximal coronary artery when compared with other segments of the coronary artery. High contrast (contrast ≥ 0.75) areas were observed in 62 of 218 segments with ≥50 % coronary artery stenosis by XCA but also in 25 of 299 segments without ≥50 % coronary stenosis.

**Conclusions:**

IR-SSFP provided good visualization of the coronary wall. This approach represents a promising noninvasive strategy for the assessment of the coronary artery wall.

## Background

The current diagnostic method of choice for the detection of coronary artery disease is conventional x-ray angiography (XCA). However, XCA provides only limited information regarding the coronary plaque burden [1]. Intravascular ultrasound (IVUS) and IVUS-based tissue characterization techniques have the ability to identify some pathological features of the atheroma [2]. However, this technique is invasive and is not appropriate for use in screening or follow-up examinations for the assessment of subclinical and advanced arteriosclerosis. Magnetic resonance imaging (MRI) with T1-weighted image is able to successfully identify coronary plaque [3–7], T1 high contrast coronary plaque was more prevalent in the context of higher-grade stenoses. Kawasaki et al. reported that hyperintense coronary plaque on non-contrast T1-weighted image was strongly associated with positive coronary remodeling, low-density findings on computed tomography (CT), and ultrasound attenuation [6]. During cardiac MRI used for functional studies and coronary angiography, steady-state free precession (SSFP) provides a high signal-to-noise ratio and high contrast-to-noise ratio with a shorter acquisition time when compared with gradient echo sequence (GRE). The contrast of SSFP depends on the T2/T1 properties of the tissue. However, using a saturation or inversion pulse combined with the SSFP sequence, predominantly T1-weighted images can be obtained [8, 9]. SSFP with a double-inversion prepulse can be performed as a segmented black-blood technique that can be used to detect the coronary artery wall [10]. However, SSFP with a single-inversion prepulse have only very infrequently been used for coronary vessel wall imaging to date. Thus, the purpose of this study was to determine whether 3D single inversion-recovery prepared SSFP (IR-SSFP) detect coronary artery wall and coronary plaque.

## Methods

### Study population

One hundred and twenty-one subjects (age, 66.2 ± 11.6 years; range, 37–86 years; 16 women) with known or suspected coronary artery disease who had undergone XCA were enrolled. The time interval between XCA and MR was 1–15 days (mean, 3 days).

Patient characteristics are summarized in Table 1.

**Table 1:** Baseline and main clinical characteristics of the patients

Age (years)	66.2 ± 11.6 (37–86)
Women : Men	16 : 105
Height (cm)	167.2 ± 10.7
Heart rate (BPM)	65.5 ± 13.0 (58–80)
Acquisition window (msec)	129.8 ± 60.1 (45–252)
Effort angina pectoris	71
Unstable angina pectoris	6
Old myocardial infarction	12
Acute myocardial infarction	31
Thoracic arterial aneurysm	1

In addition, five healthy control subjects (age, 30 ± 7 years; range, 26–38 years; five men) with no risk factors and who did not undergo XCA were enrolled. This prospective study was approved by the institutional review board of Hyogo Brain and Heart Center in September 14, 2010 and performed only after obtaining informed consent from each patient.

### X-ray angiography (XCA)

All patients underwent conventional XCA (INFX-8000 V, Toshiba Medical Systems, Tokyo, Japan) using standard techniques with multiple projections. The severity of a coronary artery stenosis was expressed as the percentage reduction in the luminal diameter, as determined using the semi-quantitative coronary analysis method. These analyses were performed according to standard algorithms to measure lesion stenosis with respect to mean reference diameter [11].

### Cardiac MR

Cardiac MR imaging was performed on a 1.5 T MR system (Intera, Philips Medical Systems, Best, The Netherlands) using a commercial five-elements cardiac synergy coil.

Subjects with a heart rate greater than 70 beats/min (BPM) received oral metoprolol (40–80 mg) 60 min before the MR examination. After a survey scan to localize the heart and diaphragm, a multi-heart phase SSFP cine sequence (TR/TE = 2.8/1.4 msec, FA = 60°, 85 heart phases, matrix = 192 x 256, SENSE factor 2) was obtained to assess the interval of minimal right coronary artery (RCA) motion for determination of the trigger delay for the subsequent coronary MRI.

Coronary bright-blood MR angiography (MRA) was performed using a previously described free-breathing electrocardiogram-triggered 3D balanced SSFP coronary MRI sequence [12–14] allowing visualization of the anatomy of the coronary artery lumen. Imaging was performed in the coronal or sagittal oblique plane.

**Fig. 1:**
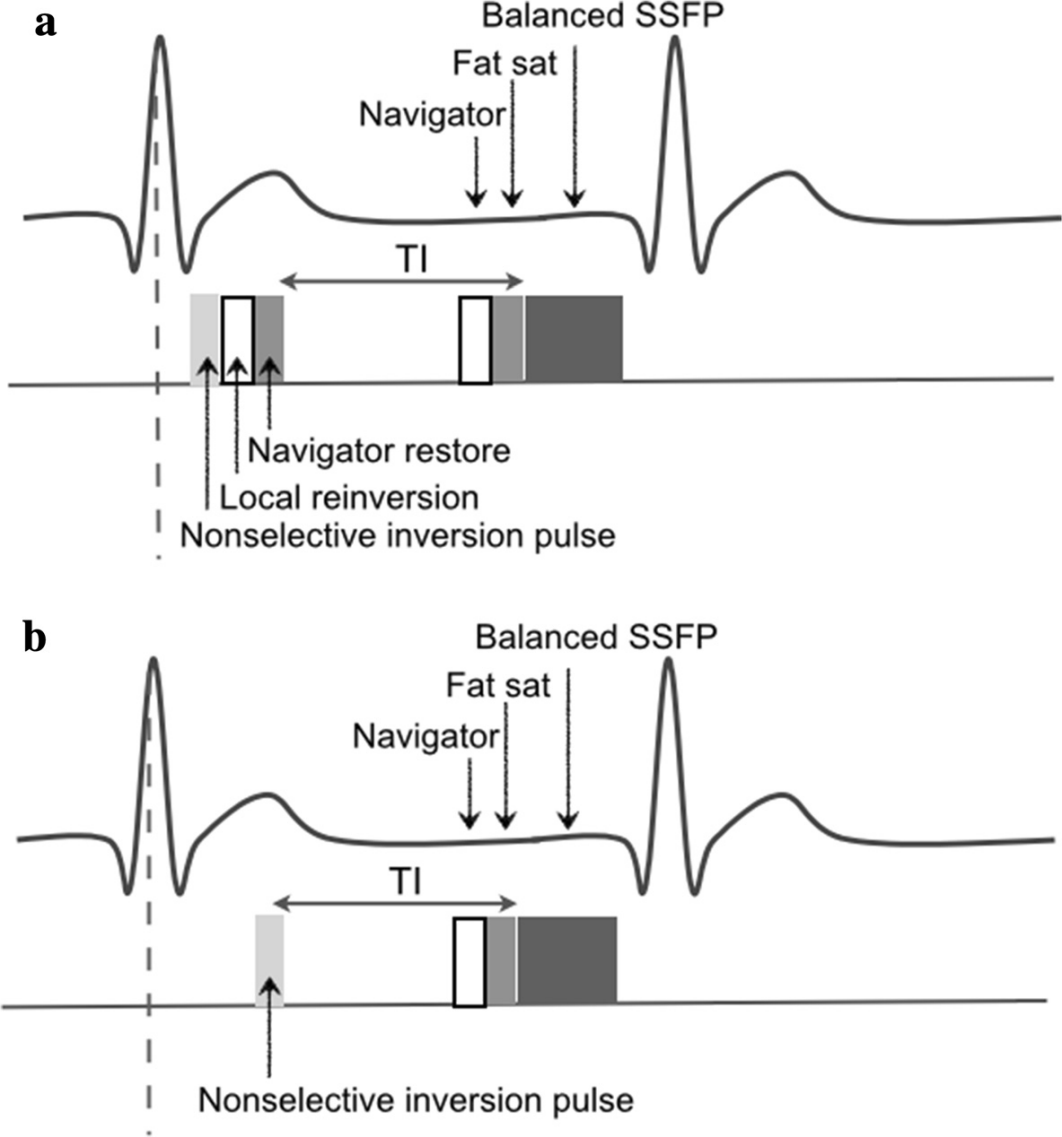
**a** Diagram of a previously described free-breathing ECG-triggered three-dimensional double inversion-recovery balanced SSFP coronary MRI sequence. **b** Diagram of a single inversion-recovery prepared SSFP (IR-SSFP) sequence

The following coronary wall MRI was a navigator-gated free- breathing and cardiac-triggered T1-weighted inversion-recovery and fat-suppressed 3D SSFP (IR-SSFP) sequence (Fig. 1). Imaging parameters included field of view = 300 mm, matrix = 304 x 512, in-plane resolution = 0.99 x 0.59 mm, slice thickness = 2.4 mm, acquisition window = 45–252 msec, TR/TE = 5.0-5.8/2.5 - 2.9 msec, FA = 90°, without SENSE, bandwidth per pixel = 781 Hz and number of slices = 20–35. In addition, a fat-saturation prepulse were used to suppress signal from the epicardial fat. The inversion time (TI) of IR-SSFP was individually assessed according to the T1 of blood using a TI scout (Look-Locker sequence) [15]. Typically, the TI adjusted to null blood was 450 msec [16]. The scanning time was about 15 min for a heart rate of 70 BPM.

### Data analysis

XCA and IR-SSFP image analyses were performed by two observers (7 and 5 years of experience in regards to MR imaging) in consensus on a segment per segment basis. Correlation between XCA and MR images was performed visually, while exact correlation between coronary bright-blood MRA and IR-SSFP images was enabled by software providing a slice registration tool within standard evaluation software (ViewForum, Philips Healthcare, Best, the Netherlands).

In each American Heart Association (AHA) coronary segment, the detection of the coronary wall (detection score, DS) was categorized into three types: score 3, good image quality with sharply defined vessel wall borders; score 2, reduced image quality but vessel wall still visible; score 1, poor image quality with poor visualization of the coronary vessel wall.

In IR-SSFP images, the mean signal intensity was measured in the coronary wall in the regions of interest (ROI) that were placed manually in the coronary wall with the highest signal intensity in the coronary segment. Contrast between the coronary vessel wall *(SIvw)* and background tissue (*SI*_BG_) was calculated for each coronary artery segment as follows [17]:

$$ contrast = \left( SIvw-S{I}_{BG}\right)/\left( SIvw + S{I}_{BG}\right) $$

where *SI*_*BG*_ refers to the signal intensity in a region of the left or right ventricle. Segments with *contrast* ≥ 0.75 were defined as “high contrast” (HC), whereas segments with *contrast* < 0.75 were defined as none.

### Statistical analysis

Results are expressed as means ± standard deviations. The relationship between IR-SSFP and the results of XCA were evaluated using Pearson’s chi-square test. Comparison of the *contrast* between the HC group and none group was made using an unpaired *t* test. All measurements were subjected to statistical analysis with statistical software (Statview version 5.0.1.2, SAS Institute). P values of less than 0.05 were considered to indicate a statistically significant difference.

## Results

**Fig. 2:**
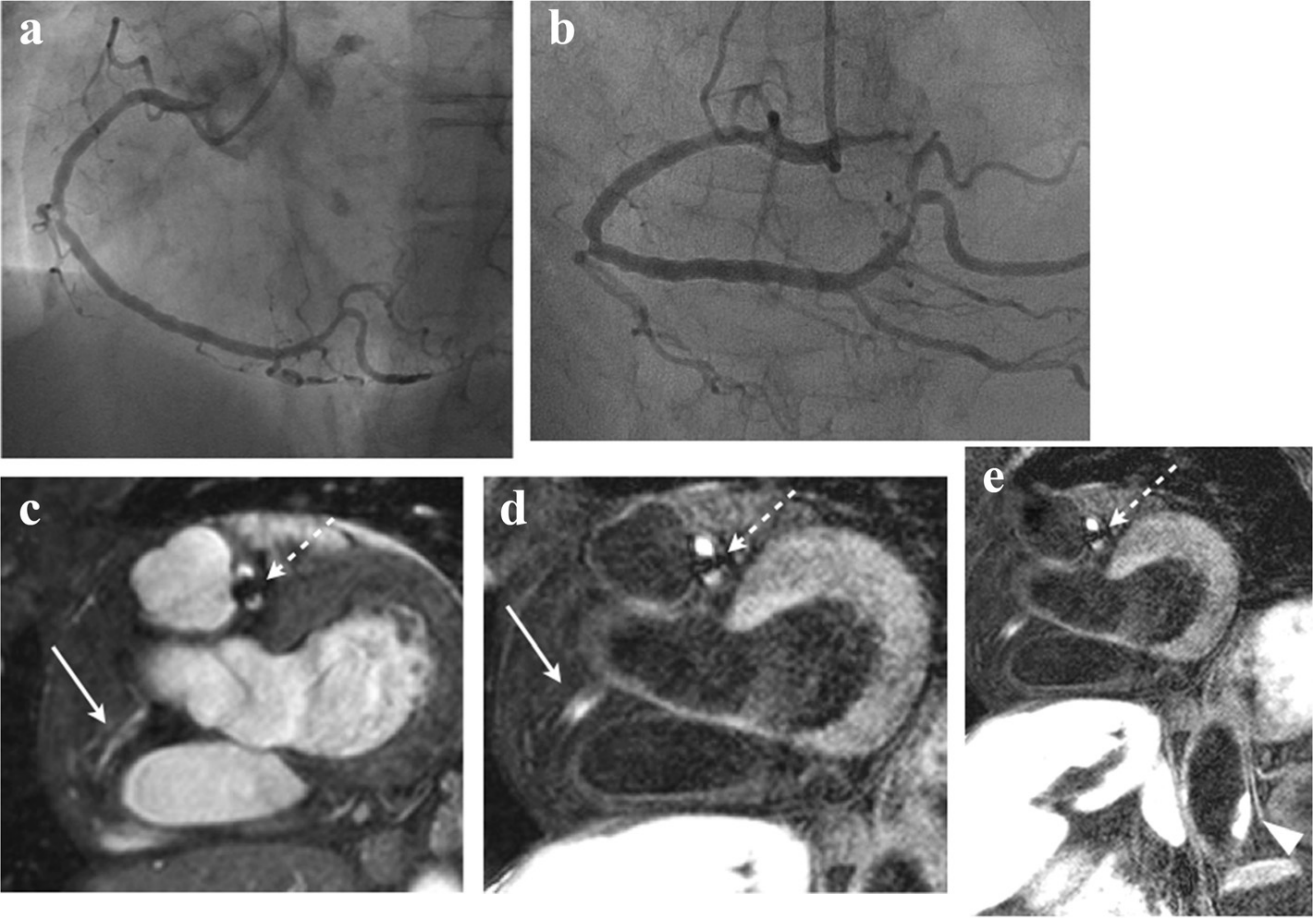
A 70-year-old patient with stents in the left anterior descending coronary artery (LAD) and left circumflex coronary artery (LCx) is shown. On the XCA (**a,b**), mild coronary stenosis was observed in the mid- right coronary artery (RCA). On the MRA(**c**), the proximal RCA was not clearly detected (arrow). On the corresponding IR-SSFP (**d**), HC can be observed in the proximal RCA (arrow). In additional, HC was observed in the abdominal aorta (**e**, arrowhead). A stent in the LAD is visible as area of signal void (dotted arrow) on MRA and IR-SSFP

**Fig. 3:**
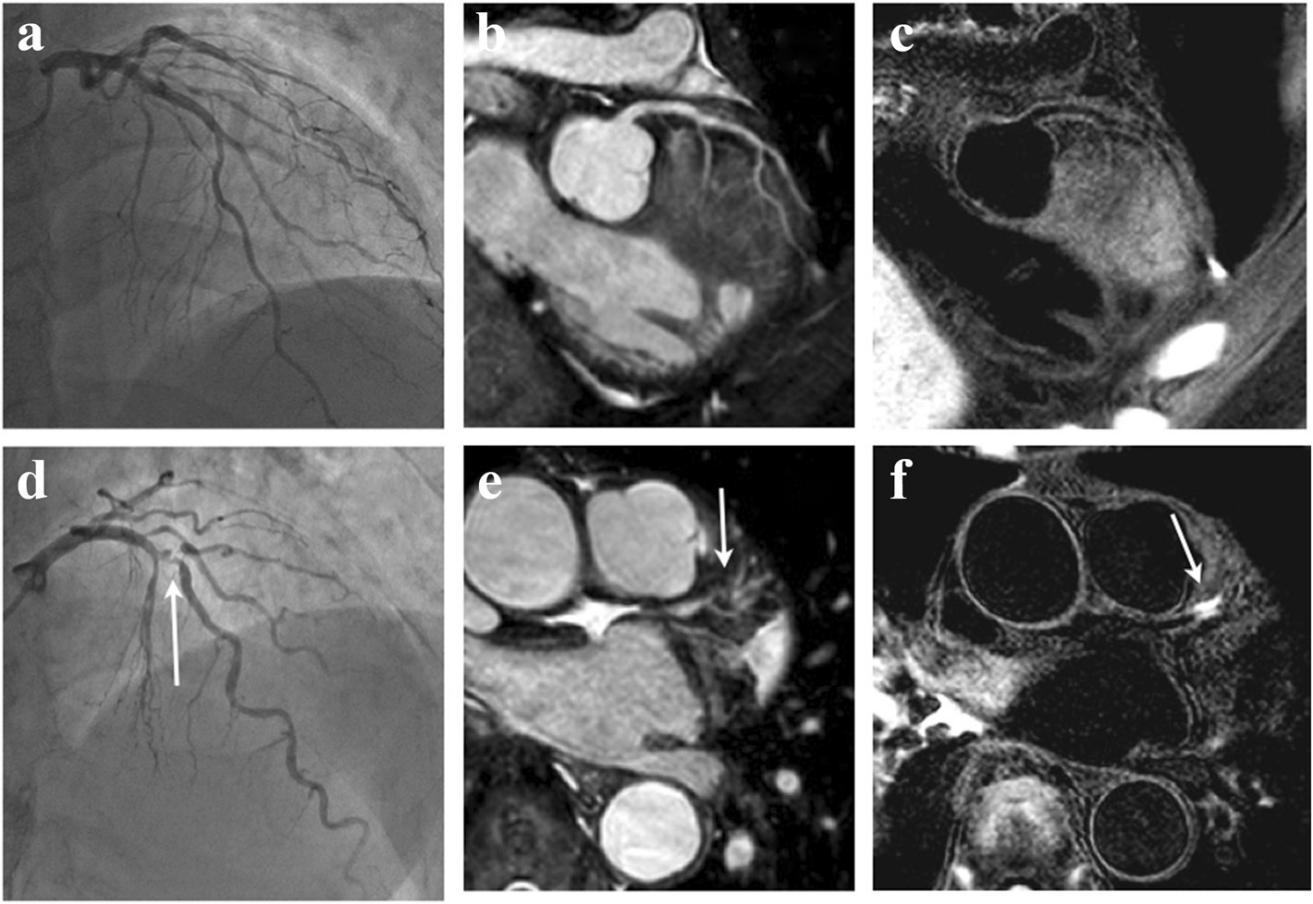
A 41-year-old patient with no coronary stenosis in the LAD is shown. On the XCA (**a**) and MRA (**b**), coronary stenosis was not observed. On the corresponding IR-SSFP (**c**), HC was not observed, and the coronary wall was clearly detected. A 81-year-old patient with severe coronary stenosis in the mid-LAD is shown. On the XCA (**d**) and MRA (**e**), severe coronary stenosis was observed (arrow). On the corresponding IR-SSFP (**f**), HC was observed (arrow)

**Fig. 4:**
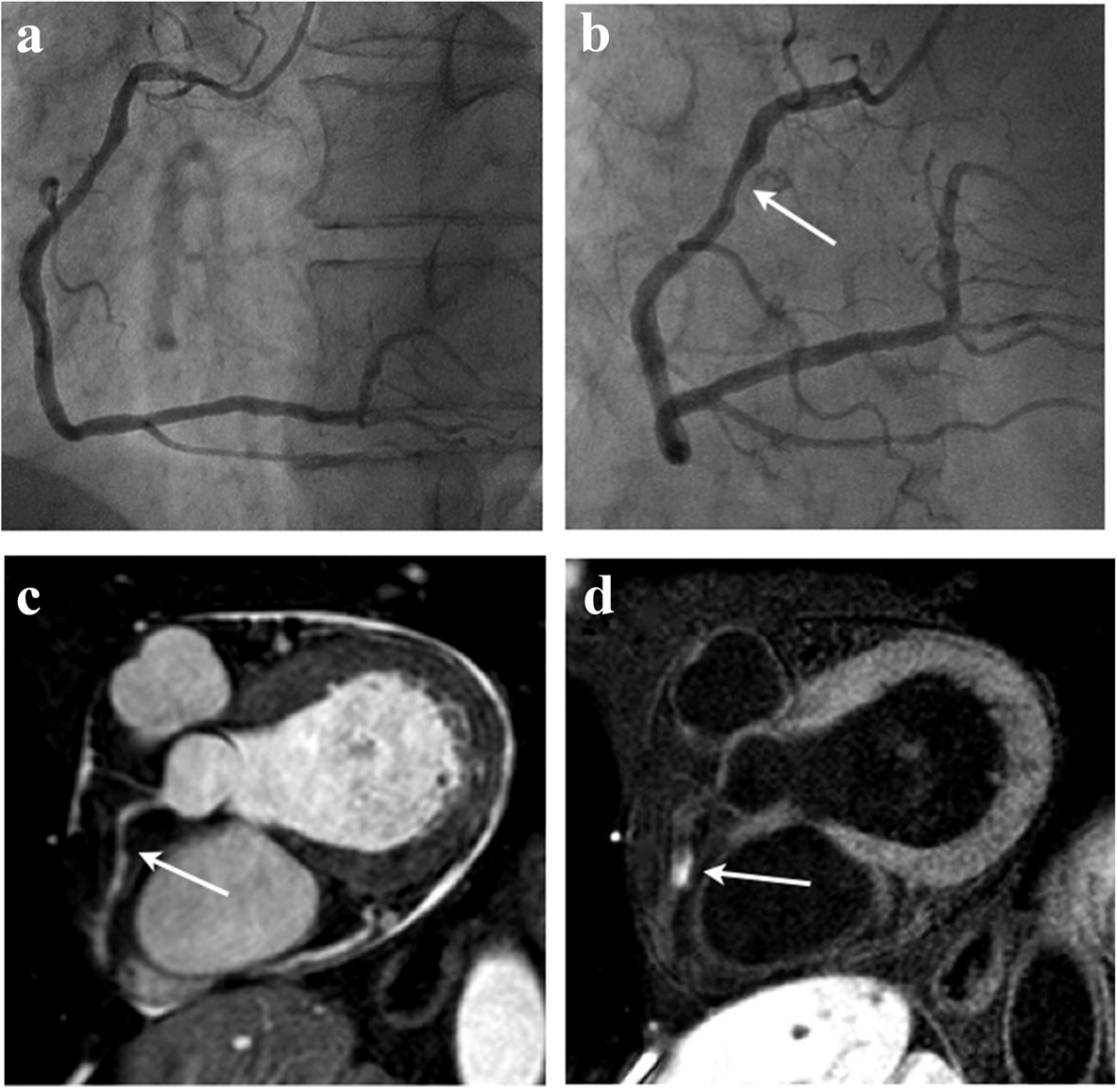
A 57-year-old patient with mild coronary stenosis in the proximal RCA is shown. On the XCA (**a,b**) and MRA(**c**), mild coronary stenosis was observed (arrow). On the corresponding IR-SSFP (**d**), HC were observed (arrow)

All patients completed cardiac MR imaging, allowing for cardiac MR imaging assessment of 517 (99 %) of 521 coronary segments in 121 subjects (four segments were not evaluated because of the presence of stents). The coronary wall was well delineated from dark-appearing blood and epicardial fat. Suppression of the blood signal was good in most subjects; 422 of 517 segments (82 %) were successfully visualized (i.e., DS = 2 or 3; Figs. 2, 3 and 4). The DS at the proximal coronary artery (segments 1, 2, 5, 6, 11) tended to be greater than at other segments of the coronary artery (Table 2). No HC was observed in the coronary wall or thoracic aortic wall in any of the healthy control subjects.

**Table 2:** Detection scores of IR-SSFP in coronary artery segment

	Number of segments		
Detect score^a^	Proximal segments *n* = 357	Others *n* = 160	Total *n* = 517
3	241	76	317
2	68	37	105
1	48	47	95

^a^3, good image quality with sharply defined vessel wall borders; 2, reduced image quality but vessel wall still visible; 1, poor image quality with poor visualization of the coronary vessel wall. The detect score at the proximal coronary artery (segments 1, 2, 5, 6, 11) tended to be greater than at other segments of the coronary artery

87 segments (17 %) were classified as HC, and 430 segments (83 %) were classified as none. The average *contrast* was significantly higher in the HC group than in none group (0.78 ± 0.03 vs. 0.48 ± 0.11, *p* < 0.001). HC were observed in 62 of 218 segments with ≥50 % coronary artery stenosis by XCA but also in 25 of 299 segments without ≥50 % coronary artery stenosis (p <0.05; Table 3).

**Table 3:** Relationship between contrast of IR-SSFP and X-ray angiographic disease

Contrast	Results of XCA	
	Segment of <50 % stenosis	Segment of ≧50 % stenosis
HC	25*	62*
None	274	156

*p < 0.05 Prevalence of HC is higher in the presence of angiographic disease

## Discussion

This study investigated whether 3D single inversion-recovery prepared SSFP can adequately characterize the coronary wall. Using inversion pulse combined with the SSFP sequence, predominantly T1-weighted images can be obtained [8, 9]. Recently, variants of SSFP have been successfully used to address myocardial late gadolinium enhancement. Inversion-recovery SSFP can be performed as a single-shot technique within a heartbeat or as a segmented technique, in a manner similar to that for segmented inversion-recovery GRE. The bandwidth for SSFP is several-fold wider than that for GRE, more lines of data can be captured in the same amount of time. Also the SSFP readout perturbs the longitudinal relaxation only slightly [9].

The drawback of SSFP is its sensitivity to off-resonance effects. These are manifest as dark bands in the image, may cause troublesome artifact. The distance between these bands is inversely related to the repetition time (TR), which is why the shortest possible TR is desirable. High-quality SSFP image requires a very short TR, a large flip angle, and a very uniform magnetic field.

In this study, several cases could not be evaluated because of the presence of stents (four segments). Coronary artery stents typically appear as well-defined areas of signal void on coronary bright-blood MRA and coronary wall imaging. Artifact size differs according to the type of stent; coronary arterial stents generate susceptibility artifacts that extend in excess of the true size of the stents and make imaging of the underlying structures impossible.

No HC was observed in the coronary wall or thoracic aortic wall in any of the healthy control subject. This negative finding is in agreement with the likely absence of coronary and aorta atherosclerosis in young subjects without cardiovascular risk factors.

HC was observed in a significantly higher proportion of coronary segments with ≥50 % stenosis than in the coronary segments without ≥50 % stenosis. Our results are in agreement with those by Oei et al. [5] and Kawasaki et al. [6] results with respect to the finding that T1 high contrast coronary plaque was more prevalent in the context of higher-grade stenoses. Kawasaki et al. reported that high contrast coronary plaque on non-contrast T1-weighted image was strongly associated with positive coronary remodeling, low-density findings on CT, and ultrasound attenuation. However, in the segment without ≥50 % coronary artery stenosis by XCA, HC on IR-SSFP were observed in 25 segments. Previous reports show that most acute coronary syndromes are caused by rupture of atherosclerotic plaques of AHA stages 3 to 4 without underlying high-grade lumen stenosis. These data suggests that high contrast coronary plaque detected by non-contrast T1-weighted imaging corresponds with a vulnerable and active coronary lesion. Early detection of this type of lesion may be beneficial in term of allowing the institution of earlier and more aggressive management [5].

Jansen et al. reported that non-contrast T1-weighted image on 3.0 T MRI has been useful for direct imaging of intracoronary thrombus in patients shortly after myocardial infarction [7]. One explanation for T1 high contrast may be the presence of intracoronary or intraplaque thrombus containing methemoglobin, which is known to have a short T1 relaxation time. Further, it may be difficult for MR signal intensity to distinguish intracoronary or intraplaque thrombus. However, we definitely state that HC demonstrates intraplaque thrombosis, because our study subjects did not have ischemic symptoms or ECG changes indicative of new ischemia.

This study possesses several notable limitations. The spatial resolution of IR-SSFP may be inadequate to image the coronary vessel wall without partial volume effects. But imaging with higher resolution would have increased scan time. As the IR-SSFP image interpretation is reduced to the detection of T1 high contrast similar to radionuclide methods, requirements on spatial resolution are less stringent [5].

We did not compare IR-SSFP with intravascular ultrasound, which is the current in vivo standard for coronary plaque assessment. However, intravascular ultrasound is an invasive technique with non-negligible risks. Furthermore, no comparison with histopathological data was performed. Another limitation of this study is the lack of follow-up imaging. Further studies, including follow-up imaging, are needed to confirm the importance of these findings.

## Conclusions

IR-SSFP provided good visualization of the coronary wall. This approach represents a promising noninvasive strategy for the assessment of the coronary artery wall.
